# Application of Genomic Technologies to the Breeding of Trees

**DOI:** 10.3389/fgene.2016.00198

**Published:** 2016-11-15

**Authors:** Maria L. Badenes, Angel Fernández i Martí, Gabino Ríos, María J. Rubio-Cabetas

**Affiliations:** ^1^Instituto Valenciano de Investigaciones AgrariasValencia, Spain; ^2^Hortofruticulture Department, Agrifood Research and Technology Centre of AragonZaragoza, Spain; ^3^Genome Center, University of California, Davis, Davis, CAUSA

**Keywords:** next generation sequencing, genome sequences, RNA-seq, association mapping, insertional mutant populations

## Abstract

The recent introduction of next generation sequencing (NGS) technologies represents a major revolution in providing new tools for identifying the genes and/or genomic intervals controlling important traits for selection in breeding programs. In perennial fruit trees with long generation times and large sizes of adult plants, the impact of these techniques is even more important. High-throughput DNA sequencing technologies have provided complete annotated sequences in many important tree species. Most of the high-throughput genotyping platforms described are being used for studies of genetic diversity and population structure. Dissection of complex traits became possible through the availability of genome sequences along with phenotypic variation data, which allow to elucidate the causative genetic differences that give rise to observed phenotypic variation. Association mapping facilitates the association between genetic markers and phenotype in unstructured and complex populations, identifying molecular markers for assisted selection and breeding. Also, genomic data provide *in silico* identification and characterization of genes and gene families related to important traits, enabling new tools for molecular marker assisted selection in tree breeding. Deep sequencing of transcriptomes is also a powerful tool for the analysis of precise expression levels of each gene in a sample. It consists in quantifying short cDNA reads, obtained by NGS technologies, in order to compare the entire transcriptomes between genotypes and environmental conditions. The miRNAs are non-coding short RNAs involved in the regulation of different physiological processes, which can be identified by high-throughput sequencing of RNA libraries obtained by reverse transcription of purified short RNAs, and by *in silico* comparison with known miRNAs from other species. All together, NGS techniques and their applications have increased the resources for plant breeding in tree species, closing the former gap of genetic tools between trees and annual species.

## Introduction

Breeding of tree species is a longer and time consuming process compared to annual species. The long generation time impairs the development of crosses until the species overcome the juvenile period, being between 3 and 10 years depending on the species. In addition, the large size of trees makes it difficult to obtain large numbers of individuals for segregation families, and precludes the management of plants under greenhouse-controlled conditions. Some otherwise useful techniques aimed at identifying gene variants such as ‘tilling’ and ‘ecotilling’ can hardly be applied to trees. Consequently, breeding by molecular marker assisted selection (“molecular breeding”) has been limited to a reduced number of traits, mainly related to disease resistance and traits with oligogenic control (Gessler and Pertot, 2012; Zuriaga et al., 2013). The identification and functional analysis of gene orthologs from model plants presents a bottleneck for efficient transformation and regeneration protocols in many of these species.

However, the availability of genome sequences along with next generation sequencing (NGS) techniques may provide new tools to overcome most of the problems faced by tree breeding. Dissection of complex traits in many important tree species has become possible through the availability of genome sequences obtained by high-throughput DNA sequencing technologies along with phenotypic variation data. Such techniques offer shortcuts for the discovery of genes linked to selected traits and simplify the analysis of diversity in a population. Association mapping (AM) and genome wide association studies (GWASs) facilitate the association between genetic markers and phenotype in unstructured and complex populations, allowing the identification of molecular markers for assisted selection and breeding. Genotyping by sequencing (GBS) (Elshire et al., 2011; Poland et al., 2012) procedures provide 1000s of markers in a population, allowing the identification of genome regions involved in traits of interest. Concerning forest tree species, characterized by large genomes such as loblolly pine, eucalyptus and oak, NGS approaches have been pivotal in providing new releases of genome sequences and improved assemblage of former ones. All together, the NGS techniques provide new tools for overcoming the long breeding process of trees and increase the breeding efficiency. In this paper we aim to review the main molecular techniques applied recently to the discovery of genes in tree species and how they can benefit the breeding programs.

## Next Generation Sequencing

Future progress in fruit tree breeding will increasingly rely on understanding the links between specific genotypes and their influence on phenotypes. In this regard, increased knowledge and availability of DNA sequences for individual cultivars will provide key information for achieving specific breeding objectives. The recent introduction of NGS technologies (Shendure and Ji, 2008; Metzker, 2010) represents a major revolution in providing new tools for marking and identifying the genes and/or genomic intervals controlling important traits for selection in breeding programs. With the ability to cost effectively produce millions of DNA sequence reads in a single run, these technologies pave the way for detailed expression analyses, mutation mapping, polymorphism discovery, studies on non-coding regions, and other uses potentially impacting the speed at which questions can be addressed and results can be accomplished in plant breeding (Mardis, 2008).

Among the different NGS platforms, Roche 454^®^ was the first commercially successful next generation system in Margulies et al. (2005). One year later, SOLiD^®^ was purchased by Applied Biosystems. In 2007, Solexa released the genome analyzer (GA), and the company was purchased by Illumina^®^. In early 2010, Illumina launched HiSeq 2000, and in the same year Ion Torrent was bought by Life Technologies^®^. These platforms employ methodologies described in the literature (Bentley et al., 2008; McKernan et al., 2009; Perkel, 2011).

Currently, third generation technologies are being introduced that streamline sequencing protocols. Helicos Heliscope^®^ (Thompson and Steinmann, 2010), Complete Genomics^®^ (Drmanac et al., 2010), Nanopore^®^ (Greninger et al., 2015) and Pacific Biosciences SMRT^®^ (Eid et al., 2009) have incorporated new modifications. First, PCR is not needed before sequencing, and secondly, the signal is captured in real time, which means that the signal is a fluorescent (Pacbio) or electric current (Nanopore) monitored during the enzymatic reaction of adding nucleotides to the complementary strand. Additionally, all of them process millions of sequence reads in parallel with very long reads, in some cases up to 10 kb long (English et al., 2012). These high-throughput DNA sequencing technologies require the development of new bioinformatic tools (algorithms and software), for storage, retrieval and analyses of huge amounts of genome-wide sequence data. Recently, the development of the newest Oxford^TM^ nanopore technology has provided novel improvements in molecular sensing such as real-time data streaming, improved simplicity, efficiency and scalability of workflows as well as direct analysis of the molecule of interest. These platforms along with the new bioinformatic tools have provided complete annotated sequences in many important horticultural crops and related wild species in a relatively short time. These sequences are stored in databases such as “*Sequence Read Archive”* from NCBI^1^, PLantGDB^2^, Phytozome^3^ and EnsemblPlants^4^.

Genome sequencing before 2010 was based on Sanger technology, which needed large DNA fragments cloned in BACs (Bacterial Artificial Chromosome). Using this technique, *Arabidopsis thaliana* was the first plant to have its genome sequenced, in 2000 by the *Arabidopsis* genome initiative. Lately, important cereal species were sequenced such as rice (*Oryza sativa* L., 389 Mb) in 2002 by IRGSP, corn (*Zea mays* L., 2300 Mb) by Schnable et al. (2009). However, bigger genomes such as oat (*Hordeum vulgare* L., 5100 Mb) and wheat (*Triticum aestivum* L., 17000 Mb), with 80–90% of repetitive sequences, were improved after NGS could be used. The genome of important species such as tomato was completed using NGS after preliminary approaches employing Sanger sequencing (Tomato Genome Consortium, 2012). Similarly, many tree species had few sequences published in databases before the appearance of second generation sequencing approaches.

Fruit tree genome assemblies have often involved a combination of NGS and Sanger technologies. Chagne (2015) reviewed the status of genome sequencing in temperate, tropical and nut tree species, with high quality genome assemblies developed mainly for diploid species. Polyploidy is a widespread feature in fruit tree species that represents a real challenge to obtain whole-genome assemblies. However, new molecular and bioinformatics methods have been developed in order to improve current genome assemblies. For instance, the TruSeq Synthetic Long Read Sequencing technology from Illumina (McCoy et al., 2014) has been utilized to assemble the polyploid sugarcane genome sequence (approximately 1 Gbp) using synthetic long reads (Riaño-Pachón et al., 2016). For this purpose, reads longer than 1.5 kbp and BLAST hits against sequences from Viridiplantae were used for genome assembly using the OLC approach (McCoy et al., 2014).

The black cottonwood (*Populus trichocarpa*) was the first perennial tree species with an available genome sequence, obtained using the Sanger technology (Tuskan et al., 2006). The first peach genome assembled (v1.0; Verde et al., 2013) was also obtained using this technology. After the introduction of NGS, the genome sequence of more species became available, and some perennial tree species were included. **Table 1** summarizes the genomes available both in fruit and in forest tree species. Former assembly of the peach genome has been improved and recently released v2.0 using NGS platforms^5^. In addition, partial sequences from cherry and almond have been made available at the *Rosaceae* Genome Database (Koepke et al., 2013^6^). Concerning forest tree species, characterized by large genomes such as loblolly pine, eucalyptus and oak, the genomes have been released recently using only NGS approaches. The sequences available are indicated in **Table 1**.

**Table 1:** Reference genome sequences of fruit and forest tree species.

Fruit species	Common names	Reference
*Carica papaya*	Papaya	Ming et al., 2008
*Malus domestica*	Apple	Velasco et al., 2010
*Theobroma cacao*	Cacao	Argout et al., 2011
*Fragaria vesca*	Strawberry	Shulaev et al., 2011
*Prunus mume*	Japanese apricot	Zhang et al., 2012
*Musa acuminata*	Banana	D’Hont et al., 2012
*Phoenix dactylifera*	Date palm	Al-Mssallem et al., 2013
*Pyrus bretschneideri*	Pear	Wu et al., 2013
*Prunus persica*	Peach	Verde et al., 2013
*Citrus sinensis*	Sweet orange	Xu et al., 2013
*Morus notabilis*	Mulberry	He et al., 2013
*Coffea canephora*	Coffee	Denoeud et al., 2014
*Elais guineensis* and *Elais oleifera*	South American oil palm	Singh et al., 2013
*Pyrus communis*	Pear	Chagné et al., 2014
*Citrus genus*	Orange	Wu G. A.et al., 2014

**Forest tree species**	**Common names**	**Reference**

*Populus trichocarpa*	Balck cottonwood	Tuskan et al., 2006
*Betula nana*	Dwarf birch	Wang et al., 2013
*Betula pendula*	Silver birch	Birchgenome.org
*Hevea brasiliensis*	Rubber tree	Rahman et al., 2013
*Picea glauca*	White spruce	Birol et al., 2013
*Picea abies*	Norway spruce	Nystedt et al., 2013
*Pinus taeda*	Loblolly pine	Neale et al., 2014
*Eucalyptus grandis*	Eucalytus	Myburg et al., 2014
*Quercus ruber*	Oak	Plomion et al., 2016

Another interesting tool that combines the use of genome sequence with identification of markers for genotyping and identification of causal mutations is the “*Genotyping by Sequencing*” technique (Elshire et al., 2011; Poland et al., 2012). GBS utilizes barcodes for the multiplex sequencing of genomic representations of different genotypes, allowing the genetic mapping in a segregant population and the identification of many SNPs. These SNPs, after a first filtering based on quality criteria, may be utilized for SNP genotyping using different technologies (Perkel, 2008; Edwards et al., 2014). GBS provides a rapid and low-cost tool to genotype breeding populations (including the two parents), allowing plant breeders to implement GWAS, genetic linkage analysis, molecular marker discovery, and genomic selection (GS) under a large scale of plant breeding programs. Additionally, GBS has been shown to be a valid tool for genomic diversity studies (Fu and Peterson, 2011). Recently this approach was reported for quantitative trait loci (QTL) analyses in peach and other species (Ward et al., 2013; Bielenberg et al., 2015). Several microarrays using the Infinium^R^ platform for SNP genotyping are available in different Rosaceae species such as peach, apple, and cherry (Chagné et al., 2012; Verde et al., 2012).

## Genome-Wide Genetic Diversity Studies

One of the main challenges in breeding is to access and use the wide genetic variation present in germplasm collections and their wild relatives. Most of the high-throughput genotyping platforms described previously are being used for studies on diversity and population structure.

Genome-wide surveys of genetic diversity are useful to elucidate the causative genetic differences that give rise to observed phenotypic variation, providing a foundation for dissecting complex traits through genome-wide association studies. The use of genome and transcriptome sequencing for SNP discovery has resulted in large SNPs collections in most crops. In diversity studies, the availability of sequences allows the identification of polymorphism rates for individual SNP markers, assisting in the selection of those SNPs with biological meaning and those that are highly polymorphic between groups and populations. The detected polymorphisms facilitate the identification of mutations related to phenotypic variation enabling a better knowledge and wider use of the diversity in breeding.

Affymetrix chips have been applied to the identification of mutations related to phenotypic variation (Hill et al., 2013). Large collections of SNPs are being validated and applied for different purposes such as map construction, map saturation, genome-wide diversity studies, and AM analyses.

In the frame of the *International Peach SNP Consortium* (IPSC), an Illumina II 9K SNP chip was specifically designed (Verde et al., 2012) and used for genotyping of variety collections of peach, cherry and almond (Romeu et al., 2014; Sánchez et al., 2014). The *RosBREED SNP Consortium* (IRSC) developed a 9K chip based on Illumina technology aimed at studying the allelic diversity of *Malus* for breeding purposes (Chagné et al., 2012).

In fruit species, the most commonly used markers for diversity studies are still simple sequence repeats (SSRs) or microsatellites. The first SSR studies in Rosaceae focused on different species from the genus *Prunus* (Dirlewanger et al., 2002; Hormaza, 2002; Aranzana et al., 2003), *Malus* (Silfverberg-Dilworth et al., 2006), and *Pyrus* (Yamamoto et al., 2002). Several local germplasm collections from temperate fruits have been studied (Bouhadida et al., 2011; Pina et al., 2014). However, in many crops progress in breeding relies on increasing the genetic variability available. Many studies of diversity are focused on the evaluation of genetic introgression from wild relatives. For instance, Richards et al. (2009) studied 20 populations of *Malus sieversii* prospected in China, concluding that diversity within each population was higher than among populations, which leads to the conclusion that this species represents a valuable source of diversity for apple breeding. In *Prunus* genus, introgressions from wild relatives resulted in gain for the commercial varieties, mainly concerning pathogen resistance (Quilot et al., 2004; Esmenjaud and Dirlewanger, 2007; Marandel et al., 2009). Other studies have focused on wild relatives from almond and apricot as a source of diversity (He et al., 2007; Zeinalabedini et al., 2008; Gross et al., 2012; Fernández i Martí et al., 2014). Increase in variability via wild relatives has been explored in other genera with relevant economic impacts such as *Citrus* (Barkley et al., 2006), and *Olea* (Belaj et al., 2010; Fernández i Martí et al., 2015a). Germplasm resources from underutilized fruits have been carried out in *Eriobotrya* (Gisbert et al., 2009) and *Diospyrus* (Naval et al., 2010).

SSRs from EST libraries were proven to be useful for genotyping diversity in fruit genera such as *Citrus* (Luro et al., 2008), *Malu*s (Gasic et al., 2009), and *Prunus* (Lazzari et al., 2008). Additionally, partial sequences of chloroplast DNA have been used for establishing phylogenetic relationships among species of different genera of fruit crops (Bausher et al., 2006; Bielsa et al., 2014).

Although SSRs are still useful markers, the trend is to develop and use SNPs. As genome sequences become available, the collection of Affymetrix chips provides large numbers of SNPs as unique markers that allow accurate identification of differences among genotypes affording many applications in genetic diversity studies, such as measuring levels of introgression among populations and targeting of specific genes and their evolution.

## Genome-Wide Sequences for Gene Mining

Among other applications, genomic data have been used for the *in silico* identification and characterization of genes and gene families related to important traits, thus enabling new tools for molecular marker assisted selection and plant breeding. Many gene families have been identified using this approach, among which are numerous transcription factor families. As an example, in *Prunus mume* MADS-box genes encoding transcription factors involved in crucial roles in plant development, especially in flower and fruit development were identified (Xu et al., 2014). In *Malus* x *domestica* the expansin gene family included 41 genes putatively related to cell-wall-loosening processes required for cell expansion and fruit softening (Zhang S.et al., 2014). The Golden Delicious apple genome was similarly mined for lipoxygenase *LOX* genes coding for enzymes that catalyze the dioxygenation of polyunsaturated fatty acids. In this study *MdLOX1a* and *MdLOX5e* were identified as candidate genes for fruit aroma volatile production in apple, based on expression and genetic evidences (Vogt et al., 2013). Mining the draft genome of papaya served to study the structure of different gene families in this species lacking recent genome-wide duplications, to accelerate the construction of physical maps of sex chromosomes and to identify a gene controlling fruit flesh color (Ming et al., 2012). The causative allele *br*, named *broomy* for pillar appearance phenotype, located previously in the linkage group 2 of the peach genetic map has been recently identified in the same region using NGS (Dardick et al., 2013). This gene, ppa010082, is an ortholog of the rice gene *TILLER ANGLE CONTROL1 (TAC1)* (Yu et al., 2007).

## Re-Sequencing Genomes

With advancements in NGS technology, whole genome re-sequencing is currently the most rapid and effective method to unravel, at the genomic level, the underlying mechanisms of species origin, development, growth, and evolution. Material from wild relatives, ancestors, landraces held in germplasm collections and modern cultivars of crop species will offer a useful gene pool to cope with existing and new breeding challenges. The main applications of genome re-sequencing include detection of genetic differences between variants, transposon fingerprinting for assessing germplasm diversity and lineages, and mapping loci associated with specific traits, such as disease resistance, fruit quality or other important agronomical traits.

To date, re-sequencing initiatives have been launched for several species including 100s of cultivars, such as the “150 Tomato Genome ReSequencing project” or “Resequencing 302 wild and cultivated accessions identifies genes related to domestication and improvement in soybean.” In the latter case, researchers were able to identify almost 10,000 new SNPs and 876,799 indels. In addition, the depth of sequencing data allowed them to identify a total of 1,614 copy number variations (CNVs) and 6,388 segmental deletions comprising 15.14 and 73.6 Mb sequences, respectively (Zhou et al., 2015). On the other hand, the 150 Tomato Genome Re-sequencing project aims to explore the genetic variation available in old breeding tomato stocks and wild tomato relatives (Aflitos et al., 2014).

Whole genome re-sequencing has been applied to most of sequenced tree genomes. In *Populus*, linkage disequilibrium studies based on genome re-sequencing suggested the feasibility of genome-wide association studies in undomesticated populations (Slavov et al., 2012). Moreover it was used to elucidate the origin of cultivated mandarin and orange trees, formed by interspecific introgressions of *Citrus maxima* into ancestral mandarin species (Wu G. A.et al., 2014). Pooled re-sequencing of 240 *Eucalyptus* genomes served to develop a 60K SNP chip suitable for different *Eucalyptus* tree species (Silva-Junior et al., 2015).

Thus, re-sequencing the genomes from multiple individuals provides an important improvement over the SNP identification procedure. The development of more comprehensive SNP and molecular marker databases will offer an extensive number of genotype markers that can be used in whole-genome linkage disequilibrium (WGLD) and candidate gene association studies (Jackson et al., 2006).

## Genome-Wide Tools for Fine Mapping and Dissection of Traits

In mapping populations, where major QTLs linked to target traits are described, resequencing individual genomes with extreme phenotypes followed by alignment of short reads against the reference genome facilitates the identification of polymorphisms associated with such traits. Using this procedure Zhebentyayeva et al. (2014) identified two dormancy associated genes *PpeDAM5* and *PpeDAM6* as strong candidates for chilling requirement and bud dormancy release in peach. Pirona et al. (2013) used two segregating populations and the Illumina 9K SNP array to redefine the map position of the fruit maturity date (MD) locus of peach. A sequence variant in the NAC gene ppa008301m co-segregated with the MD locus, suggesting this gene as a candidate for controlling fruit ripening time in peach. Vendramin et al. (2014) used a linkage map from a F2 peach progeny to map the nectarine locus in a 635 kb interval. The subsequent inspection of the genes annotated in the peach genome sequence (Peach v1.0) led to the identification of the MYB gene *PpeMYB25* as a candidate gene for trichome formation on fruit. In addition, three independent mutations in a single gene coding for a putative carotenoid cleavage dioxygenase (*PpCCD4*) were found associated with the yellow flesh trait in peach, after analyzing 37 varieties including ancestral relatives (Falchi et al., 2013).

In apple, the *Co* gene responsible for the columnar growth habit phenotype was fine mapped in a 200-kb region of the linkage group 10 (Bai et al., 2012; Moriya et al., 2012). Evidence from high throughput genomic sequencing of the ‘Wijcik’ mutant columnar habit and its wild-type ‘McIntosh’ with standard habit pointed to a 1956 bp insertion of a mobile DNA element as the likely origin of *Co* mutation (Wolters et al., 2013; Otto et al., 2014). The insertion was located in an intergenic region, but strongly affected the expression of a 2OG-Fe(II) oxygenase gene named *MdCo31*. The involvement of *MdCo31* in the columnar growth habit was functionally confirmed by the replication of a *Co*-like phenotype in *Arabidopsis thaliana* expressing constitutively this gene (Wolters et al., 2013).

In the Caucasian persimmon (*Diospyros lotus*), a conserved small RNA-dependent mechanism was found to determine sex in this dioecious plant, after high throughput genomic sequencing of sex-segregant pools of F1 plants (Akagi et al., 2014).

As an alternative to analyses in controlled crosses, AM in unstructured and complex populations is now being largely applied to many crops. Genetic resources consist of a large number of accessions with different histories, mutations, and recombination events and may represent a large reservoir of phenotypic and molecular diversity. The AM strategy has been proposed to identify polymorphisms involved in phenotypic variations and may be useful in identifying interesting alleles/traits for breeding purposes. This approach relies on the strength of association between genetic markers and phenotype. Thus, it detects and locates genes relative to an existing map of genetic markers (Mackay and Powell, 2007). AM has been successfully applied in mapping genes involved in several traits in different plant species (maize, sunflower, lettuce, potato, tomato, wheat, etc.), but only a few studies have been carried out in fruit tree crops, such as peach (Font i Forcada et al., 2013; Micheletti et al., 2015), apple (Cevik et al., 2010), pear (Oraguzie et al., 2010), almond (Font i Forcada et al., 2015a,b), and apricot (Mariette et al., 2016). In almond, these studies have been able to detect the genomic regions where candidate genes involved in the accumulation of important compounds for fruit quality such as tocopherol and phytosterol are located, using the peach reference genome. In apricot, the implementation of GWASs for Plum Pox virus (PPV) resistance utilizing NGS platforms to genotype a broad spectrum of the available apricot breeding germplasm verified a previously described single family QTL and specific candidate genes. Thus, demonstrating the utility of the combined approaches of both single family QTL and GWAS studies (Mariette et al., 2016).

## Insertional Mutant Populations

Collections of insertional mutants by T-DNA and transposon tagging have become a powerful tool for functional genomics in model plant species. In *Arabidopsis thaliana*, high-throughput procedures for plant transformation, insertion-site recovery and DNA sequencing allowed the generation of libraries of indexed T-DNA insertions at the beginning of the century (Alonso et al., 2003). They have taken advantage of sequence tagging of target genes for functional gene characterization by direct and reverse genetics approaches, providing a vast amount of information in databases and the literature. The analyses of mutant populations provide direct functional links between genes and phenotypes, help to integrate *in silico* analysis of gene and protein expression, and facilitate the association studies of natural genetic polymorphism and the phenotypic analysis of adaptation to environment. However some mutagenesis approaches cannot be applied to trees because of their outcrossing breeding systems, high heterozygosity, large body size, and long juvenile period. In contrast, gene-tagging approaches that use insertional mutagenesis to create dominant phenotypes are ideally suited for trees. The availability of poplar genome sequence allowed the first project of insertional mutations in a perennial tree species, using T-DNA activation tagging as a source of dominant and semidominant mutations (Busov et al., 2003, 2005). A dwarf poplar mutant was identified among a collection of 627 independent activation-tagged lines which contained a hyperactivated GA2-oxidase gene, leading to altered specific gibberellin contents (Busov et al., 2003). Tree stature is considered an interesting trait for breeders with potential impact on wood and fruit production and management costs. In perennial trees these studies are preliminary; however, in species with efficient transformation and regeneration protocols they may be combined with NGS technologies to constitute a valuable resource for functional genomics and breeding.

## Deep Sequencing of Transcriptomes: RNA-seq

RNA sequencing (RNA-seq) is a powerful tool for the analysis of transcriptomes due to the precise measurement of the expression level of each gene in a sample by quantifying short cDNA reads obtained by NGS technologies, allowing the comparison of entire transcriptomes between genotypes and conditions (Wang et al., 2009; Martin and Wang, 2011). RNA reads may be mapped on a reference genome or simply aligned and assembled when a sequenced genome is not available, which makes this approach suitable for any plant species.

In fruit trees, the study of transcriptomes has been widely used for characterizing pathogen infection and resistance pathways. Rodamilans et al. (2014) analyzed the hypersensitive response of plum to PPV infection. Rubio et al. (2015) evaluated the transcriptomic changes on peach leaves infected by PPV aimed at studying the plant defense response. Socquet-Juglard et al. (2013) identified genes involved in the defense response to *Xanthomonas arboricola* in peach. In these and many other studies, genes involved in cell wall metabolism, photosynthesis, hormone signaling, and plant defense mechanisms were found differentially regulated following pathogen infection.

RNA-seq was also applied to physiological issues and developmental transitions specific to perennial plants, such as bud dormancy release. Zhu et al. (2015) performed the *de novo* transcriptome assembly and expression profiling of flower buds from Chinese cherry (*Prunus pseudocerasus*). Results identified dormancy-associated MADS-box, *AGAMOUS*-like, and *APETALA3*-like as genes related to bud dormancy release. Jia et al. (2014) analyzed by RNA-seq the continuous flowering trait in longan (*Dimocarpus longan*), an evergreen subtropical species. Results identified candidate genes providing new insight into the molecular process of regulating flowering time in woody plants. Genes homologous to *SHORT VEGETATIVE PHASE* (*SVP*), *GIGANTEA* (*GI*), *F-BOX 1* (*FKF1*), and *EARLY FLOWERING 4* (*ELF4*) were found differentially expressed in cultivars flowering throughout the year and cultivars flowering only once in the season.

Other tree-specific processes approached by transcriptomic studies include wood quality traits, secondary cell wall formation and lignin synthesis, performed mostly in *Populus* (Chen et al., 2015), *Eucalyptus* (Thavamanikumar et al., 2014), *Acacia* (Wong et al., 2011), and *Tectona* (Galeano et al., 2015) genera.

Among many other studies employing RNA-seq in perennial tree species, those investigating the transcriptomic response to water deficit (Villar et al., 2011; Cossu et al., 2014; Dong et al., 2014; Behringer et al., 2015), oxidative stress (Lu et al., 2014), nutrient deficiency (Fan et al., 2014) and heat shock (Chen et al., 2014) are especially abundant and relevant under a perspective of climate change.

## Crispr/Cas9 Technology

Clustered regularly interspaced short palindromic repeats (CRISPR)/CRISPR-associated (Cas) protein 9 (CRISPR/Cas9) has emerged as an alternative to classical plant breeding and transgenic methods to improve crop plants. Until 2013, the dominant genome editing tools were zinc finger nucleases (ZFNs; Kim et al., 1996) and transcription activator-like effector nucleases (TALENs, Christian et al., 2010). Recently, CRISPR/Cas9 offered an alternative to ZFNs and TALENs for genome editing. CRISPR/Cas9 depends on small RNA for sequence-specific cleavage. Because only programmable RNA is required to generate sequence specificity, CRISPR/Cas9 is easily applicable and has developed very fast over the past year. Thus, this technique is expected to revolutionize the field of genomics. Based on the acumen of CRISPR/Cas system, it can be utilized for introducing desired changes like targeted single and multiple gene knock-outs of detrimental genes in plants (Brouns et al., 2008) and introducing SNPs into a gene of interest (Voytas, 2013) for improvement of economic traits. Until now, it has been successfully applied in many plant species such as *Arabidopsis thaliana* (Mao et al., 2013), *Oryza sativa* (Shan et al., 2013), *Sorghum bicolor* (Jiang et al., 2013), *Solanum lycopersicum* (Ito et al., 2015), *Citrus sinensis* (Jia and Wang, 2014), *Zea mays* (Svitashev et al., 2015), or *Populus tomentosa* (Fan et al., 2015).

Clustered regularly interspaced short palindromic repeats (CRISPR)/CRISPR-associated technology uses an RNA to target the Cas9 nuclease to a particular site in the genome. Cas9 unwinds the DNA at the target site and restricts both strands of the DNA, creating a double-stranded break. Lately, the break can be repaired by non-homologous end joining (NHEJ), creating a high likelihood of introducing INDELS leading to frame shift mutations in a gene or locus of interest (Nekrasov et al., 2013; Ran et al., 2013). NGS may provide the sequence data required for the selection of gene targets, and to avoid single guide RNAs (sgRNA) with a high likelihood of off-targeting. The potential off-target sequences have to be identified by searching the particular plant genome database via BLASTN against the sgRNA target site within the genes of study. Also several software tools^7^^8^ are available to predict potential off-target mutagenesis caused by Cas9/sgRNA effects for gene-editing tools. Only two studies in perennial species have applied the CRISPR/Cas9 technology, one in sweet orange (Jia and Wang, 2014), and other in *Populus tomentosa* (Fan et al., 2015); however, to date there are no studies linking NGS and CRISPR/Cas9 or showing if genetic changes induced by Cas9/sgRNA are inherited to subsequent generations in those species. Thus, the main applications of this technique are targeting food quality traits, susceptibility to pathogens or metabolomics engineering in diverting important regulatory pathways from valuable end-products (Rani et al., 2016).

## Epigenomes vs. Transcriptomes

Modulation of developmental processes by epigenetic events involving gene expression regulation by DNA and chromatin modifications persistent through repeated cycles of cell division is an emerging issue in plant science. Recently, genome wide studies of methylated DNA immunoprecipitation followed by high-throughput sequencing (MeDIP-seq) were used to characterize the DNA methylome of *Populus trichocarpa* during *in vitro* culture and plant transformation methods, in micropropagated explants, calli, and regenerated plants (Vining et al., 2013). MeDIP-seq was also employed to find tissue-specific features in *Populus* (Vining et al., 2012; Lafon-Placette et al., 2013) and developmentally regulated regions in leaves of *Eucalyptus globulus* (Hasbún et al., 2016). On the other hand, numerous chromatin modifications involving histone acetylation, methylation, phosphorylation, and ubiquitination among others can be assayed by genome wide sequencing of immunoprecipitated chromatin (ChIP-seq). In perennial trees, ChIP-seq has been used to identify dormancy-dependent H3K27 trimethylated regions in peach (de la Fuente et al., 2015) and to map trimethylated H3K4 in developing xylem of *Eucalyptus grandis* (Hussey et al., 2015).

Most interestingly, these works allowed a double assessment of gene regulation by both epigenomic (MeDIP-seq, ChIP-seq) and transcriptomic methods (RNA-seq, microarray hybridization, *in silico* data), offering a multidimensional view of the regulatory details associated with a particular process, and confirming the conservation of chromatin effects on the regulation of gene expression in the species under study.

ChIP-seq has been also utilized for the identification of binding targets of a particular transcription factor involved in cambium development and differentiation in *Populus* (Liu et al., 2015), which opens an additional way for the molecular dissection of regulatory pathways in perennial trees.

On the other hand, a previous study carried out by Fernández i Martí et al. (2015b) showed that methylation DNA may be the reason and origin of the self-compatible trait in almond. Further studies are being undertaken in other fruit tree species aimed at confirming this hypothesis.

## Genome-Wide Identification of microRNAs

MicroRNAs (*miRNAs*) are non-coding short RNAs involved in the regulation of different physiological processes through modification of the stability of complementary target transcripts. They can be identified by high-throughput sequencing of RNA libraries obtained by reverse transcription of purified short RNAs, and by *in silico* comparison with known miRNAs from other species. Numerous miRNA searches have been performed in perennial trees such as olive, persimmon, citrus, grapevine and peach, among others. In olive, a comprehensive study described miRNAs related to alternate bearing and fruit development. The differential accumulation of miRNAs under different developmental phases and tissues indicated that control of nutrition and hormone, together with flowering processes had a noteworthy impact on alternate bearing in olive (Yanik et al., 2013). The miRNA profiling at different stages of fruit development in pear allowed the identification of factors involved in fruit development and fruit quality through the regulation of lignin synthesis, sugar and acid metabolism and hormone signaling pathways (Wu J.et al., 2014). In persimmon, the identification of miRNAs involved in regulation of proanthocyanidin biosynthesis added valuable information on the mechanisms of natural astringency removal (Luo et al., 2015). The comparative profiling of miRNAs between red-flesh mutant and wild type sweet orange contributed to the identification of miRNA-mediated molecular processes regulating lycopene accumulation in sweet orange (Xu et al., 2010). In addition, novel cold responsive (Zhang X.-N.et al., 2014) and early flowering (Sun et al., 2012) microRNAs were identified in *Poncirus trifoliata*. Deep sequencing has been also used to identify miRNAs in grapevine (Pantaleo et al., 2010) and apple (Xia et al., 2012). In peach, the analysis of miRNA accumulation served to identify miRNA related to drought and chilling stresses (Barakat et al., 2012; Eldem et al., 2012).

In apple the columnar apple phenotype is connected to gene regulation by miRNAs, since transgenic apple expressing the *MdDRB1* (*Double Stranded RNA Binding Protein*) gene, associated with the biogenesis of miRNA, produces a phenotype similar to the columnar apple type of ‘Wijcik’ mutant (You et al., 2014).

## Impact of NGS on Breeding

All the techniques described are providing new tools that overcome most of the problems faced in tree breeding. There are many examples of how these new tools benefit tree breeding. To describe the current and future impact in specific breeding programs we reviewed the impact in peach. We selected this species because among the temperate fruit crops, peach is the third most important in the world after apples and pears. The breeding activity in this species is very active; more than 100 new cultivars are released per year (Strada and Fideghelli, 2003). Peach has a small genome and relatively short generation time, around 3 years, becoming the model for fruit tree species. This species summarizes both: high breeding and molecular activity. In this section we review some examples in which the NGS techniques have been applied in peach breeding and how in recent years they are generating results that will change the procedures of selection in the current breeding programs.

The peach genome sequence was available in Verde et al. (2013). The availability of a complete peach genome assembly combined with data from a long history of breeding, including several peach maps from families segregating for important traits and sets of phenotypic data, provide new opportunities for use of genomic and phenotypic data; all together will increase the power of the classical breeding.

Fruit quality traits showing quantitative inheritance are among the most important traits used in peach breeding. In the past, they have been difficult for phenotypic screening and needed large progenies. Martínez-García et al. (2013) built a high-density SNP map, based on whole-genome sequencing. They detected significant QTLs and candidate genes for quality traits related to chilling injury. Fruit ripening or MD is an important trait for lengthening the peach season. Fine mapping and bioinformatic analysis of the genome sequence associated with the MD locus allowed the identification of a sequence variant co-segregating with the MD trait. This variant provides potential marker-assisted breeding of new cultivars differing in MD. Another important fruit quality trait is resistance to cold storage, slow ripening (SR) is an interesting trait for selecting this resistance. Whole genome analysis allowed the identification of a deletion co-localizing with the SR trait (Nuñez-Lillo et al., 2015). Fruit acidity is a major determinant of fruit quality in peach. Wang et al. (2016) used genome-wide association studies by re-sequencing 129 varieties to identify a SNP linked to the acidity trait. Genotyping of a collection of 436 varieties served to verify this marker, thus enabling marker assisted selection of non-acid genotypes. The nectarine trait is due to a mutation resulting in a glabrous phenotype. Analysis of genomic re-sequencing data from peach/nectarine accessions pointed to an insertion of a LTR retroelement as the likely cause of the nectarine phenotype (Vendramin et al., 2014). Red color of flesh is due to high anthocyanin content leading to antioxidant properties that are attractive to consumers, and consequently has become an interesting trait in many breeding programs. Mapping projects combined with whole genome sequencing identified three genes of the dihydroflavonol-4-reductase family as good candidates for the control of this trait (Shen et al., 2013).

Similarly to fruit quality, high-throughput sequencing helped to dissect complex traits and to identify candidate genes for other traits such as adaptability to chilling (Bielenberg et al., 2015), and resistance to brown rot (Martínez-García et al., 2013), bacterial spot (Yang et al., 2013), and PPV (Zuriaga et al., 2013).

In all cited cases, NGS played an important role in the identification of gene candidates and allelic variants responsible for complex traits, allowing more efficient screening procedures of such traits by molecular markers developed from those allele variants and/or genes. The information emerging is changing breeding procedures very rapidly.

## Conclusion

The profusion of recent NGS-based studies performed in trees, briefly outlined in this review, firstly indicates that the old promise of genomics to make gene approaches affordable to many non-model species is already a matter of fact, driven by technological and important economic improvements. In fact, DNA sequencing cost based on calculations performed by the National Human Genome Research Institute has decreased from about $5,000 per raw megabase in 2001 to $0.015 in 2015^9^. While the task to identify genes related to a given biological process in any plant species has become easier and cheaper, publication requirements are increasingly exigent. For processes specific to tree species, a simple list of identified transcripts was often considered as a sufficient contribution to the basic knowledge of its regulation; however, currently, additional experimental evidences are expected to support genomic data. Thus, advanced resources are required in order to go one step further, or to tackle issues with wider interest. Such methodologies should optimally offer functional information of genes identified by means of biochemical analysis, genetic manipulation of gene expression and gene disruption or editing using the CRISPR/Cas9 or related systems. A sustained advance of tree science relies eventually on the use of these and other forthcoming methodologies capable of bridging the gap with standard model species.

## Author Contributions

MB and MR-C revised the sections of NGS, Genome wide association, Genome-wide tools for fine mapping and dissection of traits. GR revised Insertional mutant populations, Deep sequencing of transcriptomes: RNA-seq. AF revised Genome-wide identification of microRNAs. All authors revised the final version of the MS and agree with the content.

## Conflict of Interest Statement

The authors declare that the research was conducted in the absence of any commercial or financial relationships that could be construed as a potential conflict of interest.

## Footnotes

1 http://www.ncbi.nlm.nih.gov/sra

2 http://www.plantgdb.org

3 http://www.phytozome.net

4 http://plants.ensembl.org/index.html

5 https://www.rosaceae.org/species/prunus_persica/genome_v2.0.a1

6 http://www.rosaceae.org

7 http://chopchop.cbu.uib.no

8 http://cbi.hzau.edu.cn/crispr/

9 https://www.genome.gov/sequencingcostsdata/
